# From Perceived Economic Inequality to Support for Redistribution: The Role of Meritocracy Perception

**DOI:** 10.5334/irsp.1013

**Published:** 2025-04-02

**Authors:** Lope Tejero-Peregrina, Guillermo Willis, Ángel Sánchez-Rodríguez, Rosa Rodríguez-Bailón

**Affiliations:** 1 Universidad de Granada, ES; 2 University of Granada, ES; 3 University of Salamanca, ES

**Keywords:** Perceived Economic Inequality, Descriptive Meritocracy, Attitudes towards Redistribution, Normative Climate

## Abstract

Economic inequality negatively impacts the welfare in our societies, yet there is reluctance to support measures aimed at alleviating its effects. To enhance our comprehension of how inequality influences support for redistribution, this paper investigates the mediating role of descriptive meritocracy (i.e., the degree to which meritocracy is perceived to exist in society). Using a cross-sectional study (N = 1536) and a follow-up experimental-causal-chain design in two subsequent experiments (N = 530), we show that the perception of inequality leads to the perception that society is not meritocratic, which, in turn, promotes support for redistribution. These results underscore the significance of perceiving economic inequality in order to dismantle the normative meritocratic narratives that hinder its reduction. We discuss these findings as part of the effects of economic inequality on the normative climate that influences our individual outcomes.

## Introduction

Just 2% of the overall wealth is accrued by the bottom 50% of individuals, while the upper 10% echelon of society possesses three-quarters of the world’s wealth (Chancel et al. 2022). Given the global high rates of economic inequality, it is not surprising that concern has grown about the psychological and social repercussions of its effects (Buttrick et al. 2017; Peters & Jetten 2023; Rodríguez-Bailón 2020; Wilkinson & Pickett 2009; Wilkinson & Pickett 2017), and the focus on the attitudes towards actions aimed at its mitigation (Dallinger 2010; García-Sánchez et al. 2018; Schmidt-Catran 2016; Wienk et al. 2021).

Redistribution of wealth has demonstrated efficacy in ameliorating economic disparities (Atkinson 2015; Chancel et al. 2022). According to Meltzer & Richard’s model (1981), one would anticipate an escalation in demands for redistribution as economic inequality increases. However, redistribution is not as popular as one could expect given the high rates of global inequality (Arsenio 2018; Son Hing et al. 2019). Scholars proposed that individuals’ perceptions of economic inequality, rather than actual inequality (Willis et al. 2022), and beliefs concerning inequality should be considered to understand the relation between inequality and motivations towards redistribution (García-Castro et al. 2020; García-Sánchez et al. 2020). Notably, empirical evidence has shown that perceiving inequality, particularly in daily life, fosters support for redistribution (García-Castro et al. 2020). Furthermore, the association between perceived inequality and support for redistribution has been observed to be mediated by intolerance of inequality (García-Castro et al. 2021) and moderated by system-justifying beliefs (García-Sánchez et al. 2018; Rodriguez-Bailon et al. 2017).

Despite these insights, a critical gap remains in understanding the specific normative processes that mediate the impact of perceived economic inequality on individual outcomes, especially attitudes towards redistribution. By normative processes, we mean the perception of social norms as mechanisms that explain the effect of perceived inequality. This gap suggests the need for a more nuanced exploration of the mechanisms through which perceptions of inequality shape redistributive preferences. Building on the psychosocial approach to economic inequality (Wilkinson & Pickett 2009), Sánchez-Rodríguez et al. (2023) proposed the Economic Inequality as Normative Information Model (EINIM) to shed light on how the perception of inequality affects our attitudes and behaviors through the normative climate—i.e., the set of features that individuals perceive as generalized within a social environment—that unequal settings give rise to. The model posits that the degree of perceived economic inequality allows us to infer the social norms that operate in a given context. Consequently, consistent with the descriptive norms effect (Cialdini et al. 1991), individuals may be inclined to adopt attitudes and exhibit behaviors that align with this inferred normative climate. In essence, the way perceived inequality impacts individual outcomes may be mediated by these norms.

We believe that a crucial aspect of this normative climate involves societal beliefs about fairness in the distribution of rewards, namely perceptions of meritocracy. As such, the present research seeks to examine the role of descriptive meritocracy, which pertains to the perception that individuals are rewarded based on their efforts and talent (Castillo et al. 2019; Son Hing et al. 2011), as a normative process that may explain the impact of economic inequality on redistribution. Particularly, we aimed to analyze whether the perception of economic inequality influences perceived meritocratic societal norms, which in turn can affect preferences for redistribution.

### Economic Inequality and Descriptive Meritocracy

While some studies indicate a positive association between objective economic inequality and meritocracy (Mijs 2021), the evidence is not straightforward (Bartram 2023; Morris et al. 2022).

Mijs (2021) found evidence that in societies with greater economic inequality, individuals tend to endorse meritocratic beliefs more strongly. According to the author, this could be because, in contexts of high inequality, people might rationalize disparities by attributing success to individual effort and talent, thus justifying the status quo and reducing cognitive dissonance regarding the presence of inequality.

However, other studies have challenged this positive correlation. For instance, Bartram (2023) argues that the relationship between objective economic inequality and meritocratic beliefs can vary significantly depending on other contextual factors such as cultural norms, political climate, and historical background. Similarly, Morris et al. (2022) showed that there is no robust relationship between local income inequality and meritocratic beliefs in England. These studies suggest that in some societies, high levels of economic inequality might lead to questioning the meritocratic principles.

In summary, the effect that inequality, measured through economic indicators, has on meritocratic beliefs is hardly conclusive. We argue that it is more pertinent to examine how perceived inequality, rather than its objective counterpart, intersects with meritocracy (Willis et al. 2022). This choice is motivated by two key arguments.

First, the correlation between objective and perceived inequality is relatively weak (Sprong et al. 2019), indicating that individuals’ perceptions of inequality do not always align with actual economic disparities. This discrepancy underscores the importance of focusing on subjective experiences and perceptions, as these have a more direct impact on individuals’ attitudes and behaviors than economic indicators (García-Castro et al. 2022).

Second, according to the model on which we build our research, the EINIM, it is the perceived level of economic inequality that leads individuals to infer a particular normative climate in society, rather than actual inequality, per se. Thus, we suggest that it will be the perception of inequality, rather than inequality measured with economic indicators, that most accurately and consistently shapes the descriptive meritocratic norm we infer from economically unequal contexts.

### Perceived Economic Inequality and Descriptive Meritocracy

Previous research has shown that perceived economic inequality shapes the descriptive norms of society (Sánchez-Rodríguez et al. 2023). For instance, when exposed to heightened levels of economic inequality, people seem to infer others as more competitive and individualistic (Cheng et al. 2021; Sánchez-Rodríguez et al. 2019; Sommet & Elliot 2022), less communal (Moreno-Bella et al., 2022), and more worried about their social status (Melita et al. 2021). In sum, these studies demonstrate the relationship between the perception of economic inequality and the emergence of distinct norms in society.

We contend that the normative inferences identified in prior research constitute only a subset of the broader psychological responses elicited by economic inequality. Beyond these norms, individuals also form meritocracy-related inferences derived from unequal contexts. In this line, for example, the literature points to the fact that the expectation that people can improve their position on the social ladder weakens as perceived economic inequality rises (Browman et al. 2019; McCall et al. 2017). Recent empirical evidence by Zhu et al. (2022) further supports this idea, demonstrating that perceptions of economic inequality predict the beliefs that society is less meritocratic. According to their results, perceiving the distribution of resources as unfair explains how inequality diminishes the expectation that hard work is rewarded in society.

In light of these findings, one could predict that the normative climate inferred from the exposure to high levels of economic inequality will be of low meritocracy. Upon establishing the validity of this assumption, it raises the question of how such a low meritocratic climate would influence individuals’ attitudes towards redistribution.

### Descriptive Meritocracy and Attitudes towards Redistribution

As argued by Madeira et al. (2019), meritocracy operates as two distinct, yet interconnected, mechanisms: first, as a social equalizer, allowing individuals to improve their socioeconomic status; and second, as a legitimizing force, providing a socially acceptable justification for individuals’ position (e.g., Levy et al. 2006). By asserting that socioeconomic status is determined by individual merits, such as effort or talent, descriptive meritocracy serves as a rationale for the unequal distribution of wealth. Moreover, it cultivates the conviction that success can be attained by people who work hard and/or display the requisite skills, excluding the impact of uncontrollable factors such as race, gender, or social class. As a result, individuals who perceive society as meritocratic tend to oppose government intervention in the distribution of wealth, as they think that the system is already fair and no additional measures are needed (Kudrnáč & Petrúšek 2023).

Moreover, previous findings showed that beliefs that meritocracy exists in society act as a significant impediment to garnering individual support for redistributive policies (Alesina et al. 2018; Alesina & Angeletos 2005; García-Sánchez et al. 2020; Hartwich & Becker 2023). From this perspective, there should be a negative and causal relationship between perceiving that there is a meritocracy (i.e., descriptive meritocratic norms) and support for redistributive policies.

In summary, the perception of economic inequality may impact the extent to which the meritocratic norm is perceived within society, which, in turn, may influence individuals’ attitudes towards redistribution. Hence, we hypothesized that the descriptive meritocratic norm may serve as an explanatory mechanism for understanding the impact of perceived economic inequality on support for redistribution.

### The Current Research

**Figure 1:**
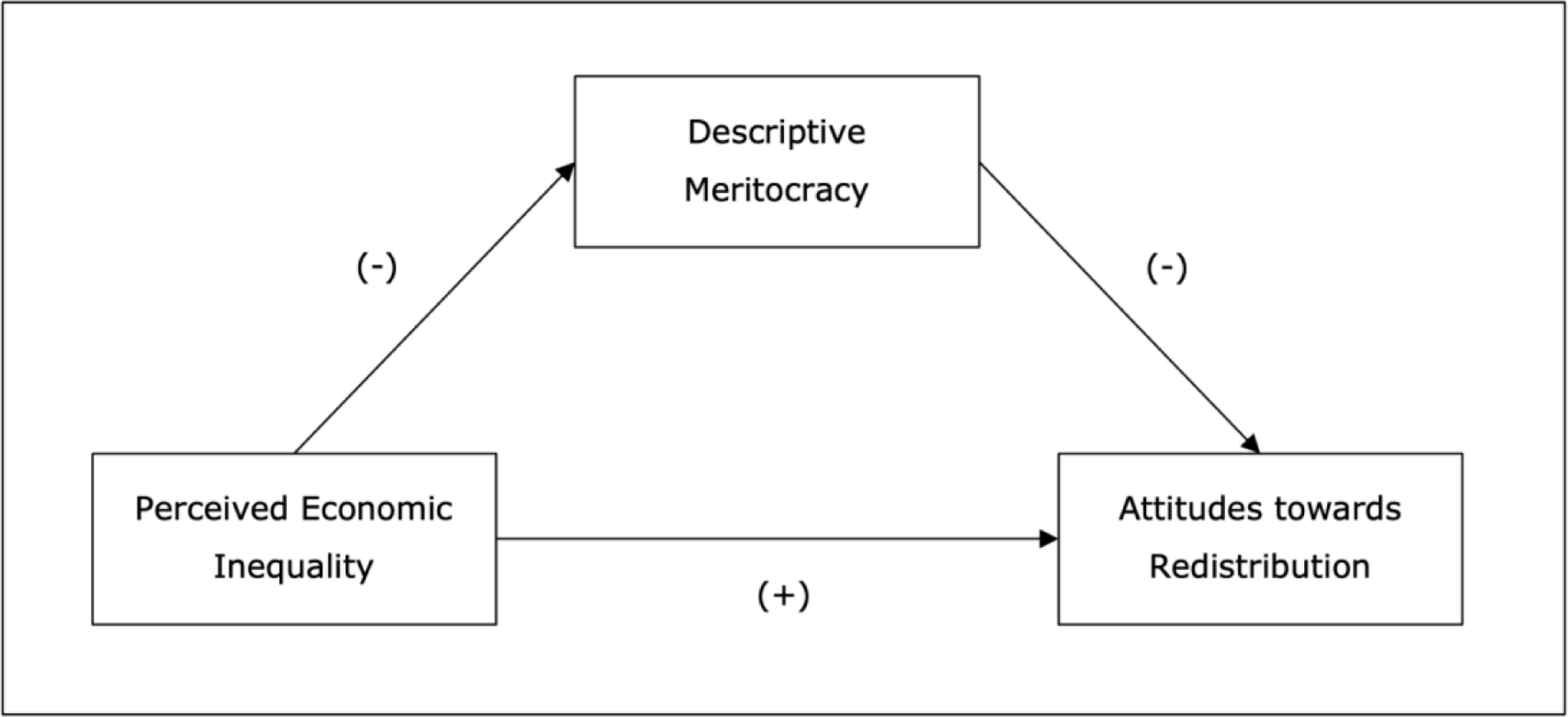
Hypothesized model in current research.

As our main hypothesis, we predict that the perception of inequality will lead to the perception that society is not meritocratic, which in turn will promote support for redistribution (see Figure 1).

Through three studies, a cross-sectional study with a stratified quota-based sample (*N* = 1536) and two experiments (*N* = 530), we test the hypothesized relationship between perceived economic inequality and attitudes towards redistribution through descriptive meritocracy. Study 1 tests the proposed mediation with a measurement-of-mediation design. To experimentally replicate the indirect effect, we then follow a sequential experimental-causal-chain design (Spencer et al. 2005): Experiment 2a tests the causal effect of perceived economic inequality on descriptive meritocracy, and Experiment 2b investigates the causal effect of descriptive meritocracy on attitudes towards redistribution.

Moreover, given that perceptions of meritocracy and support for redistribution in society may vary according to subjective socioeconomic status and political orientation (Brown-Iannuzzi et al. 2015; Brown-Iannuzzi et al. 2017; Dawtry et al. 2015; Weiner et al. 2011; Zimmerman & Reyna 2013), we included them as control variables in our analyses. Furthermore, considering that perceptions of meritocracy (i.e., descriptive meritocracy) and preferences for meritocracy are two close but independent constructs (Castillo et al. 2021; Son Hing et al. 2011; Zimmerman & Reyna 2013), we will include the latter as control in our analyses too.

We report all manipulations, measures, and exclusions in these studies. The data, code, and Supplemental Materials are available at: https://osf.io/7zh9w/?view_only=cac47b5f0d0f4db49a56a4d10759baf6.

## Study 1. Cross-sectional study

### Methods

#### Participants and procedure

We collected data for this study using the services of the NETQUEST company. To mirror the distribution of the Spanish population as stated by the *Instituto Nacional de Estadística* (National Institute of Statistics) of Spain, this sample was stratified using quotas based on gender, age, social class, and region of residence as established by the Nielsen standards (for frequencies analysis see pp. 5–6 in the Supplementary material). Respondents who failed more than one (out of three) attention checks during data collection were screened out of the sample. This study was not preregistered.

The final sample size was 1,536 (*M_age_* = 48.41, *SD_age_* = 17.21; Women = 51.43%). We conducted a sensitivity analysis using G*Power (Faul et al., 2009), which revealed that our sample size could detect an effect size for a correlation of *r* ≥ .07 (*α =* .05*, power =* 80%).

#### Measures

**Province Income Inequality**. We used the 2020 province-level Gini coefficient from the National Institute of Statistics of Spain. The index reports how income is distributed across the population in 51 provinces by comparing the proportion of people ranked by income to the proportion of total income they receive. It is a measure of inequality that takes the value 0 in case of perfect equality and the value 100 in case of perfect inequality.

**Perceived Economic Inequality**. We used two items: ‘To what extent do you think that the distribution of the resources in Spain is unequal?’ and ‘To what extent do you think that the distribution of the resources in Spain is equal?’ (Sánchez-Rodríguez et al. 2019). Answers were provided on a 7-point Likert scale ranging from 1 (*Not at all*) to 7 (*Completely*). We reversed the scores on the second question and averaged the two questions (*Spearman-Brown* ρ = .66, Eisinga et al. 2013). Higher scores indicate high perceived economic inequality (*M* = 5.48, *SD =* 1.16).

**Descriptive Meritocracy**. We used two items adapted from the meritocracy scale of Castillo et al. (2019) (‘In Spain people are rewarded for their efforts’ and ‘In Spain people get what they deserve’). Answers were provided on a 7-point Likert scale ranging from 1 (*Totally disagree*) to 7 (*Totally agree*). We computed the mean score of these items (*Spearman-Brown* ρ = .77). Higher scores indicate high perceived meritocracy (*M* = 2.98, *SD =* 1.34).

**Attitudes Towards Redistribution**. We used a 7-item scale adapted from García-Sánchez et al. (2022) (e.g., ‘The government has a responsibility to reduce the income gap between those who have more and those who have less’). Answers were provided on a 7-point Likert scale ranging from 1 (*Totally disagree*) to 7 (*Totally agree*). We computed the mean score of these items. Higher scores indicate more positive attitudes towards redistribution (*α* = .87, *M* = 5.22, *SD =* 1.26).

We also measured subjective socioeconomic status (from 1 = *The worst off* to 10 = *The best off*; Adler et al. 2000), political orientation (from 0 = *Left-wing* to 10 = *Right-wing*), and preferences for meritocracy with two items adapted from Castillo et al. (2019) (e.g., ‘People who work hard deserve to earn more than those who do not’, from 1 = *Totally disagree* to 7 = *Totally agree*).^1^

### Results

Table 1 presents the descriptive statistics and Pearson correlations between the variables included in this study.

**Table 1:** Descriptive statistics and Pearson correlations between variables.


	MEAN (SD)	1	2	3	4	5	6	7

1. Province Income Inequality	32.34 (2.04)							

2. Perceived Economic Inequality	5.48 (1.16)	–.04						

3. Descriptive Meritocracy	2.98 (1.34)	.08**	–.31***					

4. Attitudes towards Redistribution	5.22 (1.26)	–.01	.32***	–.21***				

5. Preferences for Meritocracy	5.47 (1.15)	.02	–.08**	.12***	–.20***			

6. Subjective Socioeconomic Status	5.16 (1.56)	.06*	–.15***	.28***	–.18***	.09***		

7. Political Orientation	4.17 (2.67)	.05	–.21***	.19***	–.45***	.32***	.09***	

8. Age	48.41 (17.21)	.04	.01	.11***	.09***	.16***	.11***	.11***


Note: ***p < 0.001, **p < 0.01, *p < 0.05.

#### Preliminary Analyses: Actual vs. perceived inequality on descriptive meritocracy

Prior to proceeding with the main analyses, we examined the appropriateness of using perceived inequality, rather than inequality measured with the Gini, as the main predictor in our model. To do this, we tested the two reasons proposed in the introduction.

First, in the correlation analysis (Table 1), we found that the relationship between economic inequality measured with the Gini and perceived inequality is weak, negative, and not significant (*r* = –.04, *p* = .09).

Second, both perceived inequality (*b* = –.36, *SE* = .027, *p* < .001) and inequality measure with the Gini (*b* = .05, *SE* = .016, *p* = .005) were significantly associated with descriptive meritocracy. However, the Wald test revealed that the difference between the effects of perceived inequality and actual inequality on descriptive meritocracy was significant, *F*(1,1533) = 164.5, *p* < .001, *η_p_^2^* = .10, suggesting that the former is significantly stronger than the latter.

The confirmation of the two arguments supports the consideration of perceived inequality as the main predictor of the model.

#### Main Analysis: The effect of perceived economic inequality on attitudes towards redistribution through descriptive meritocracy

**Figure 2:**
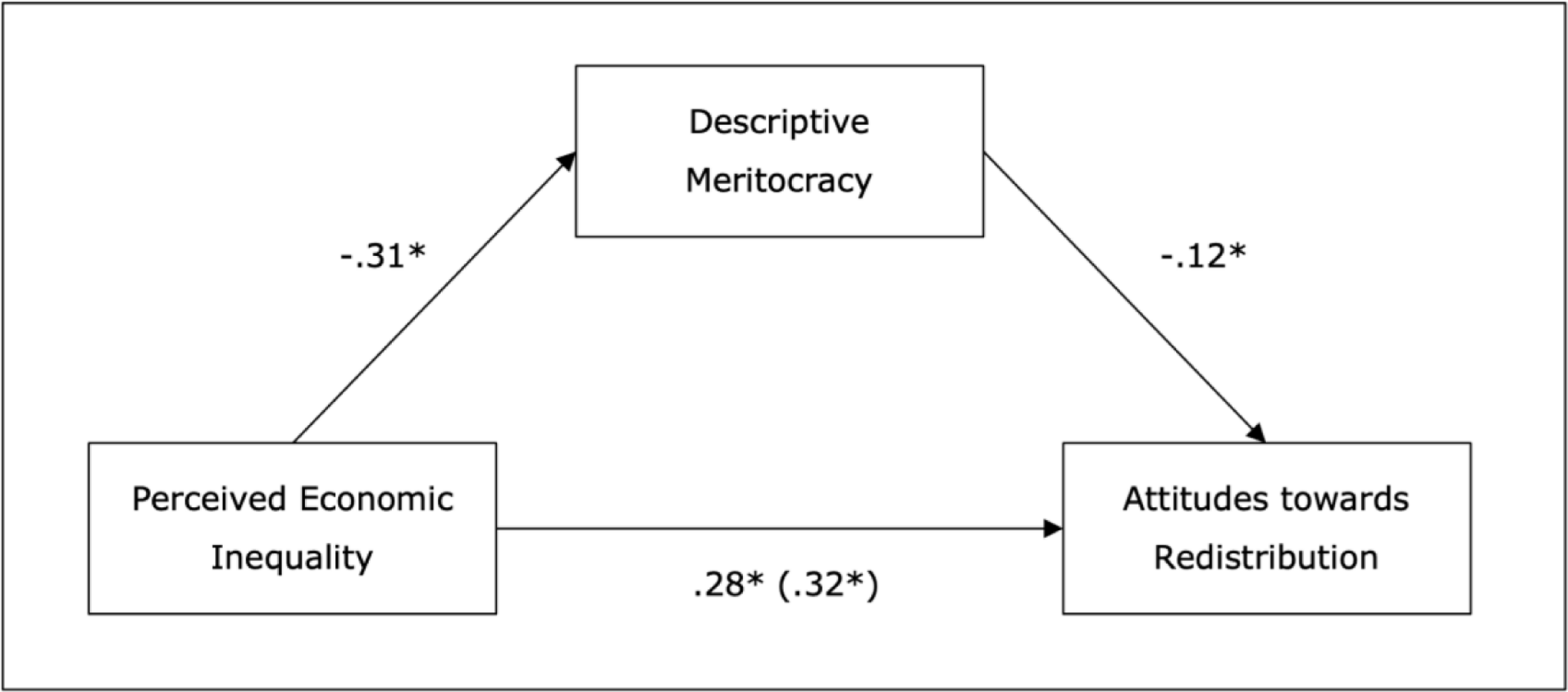
Indirect effect of perceived economic inequality on attitudes towards redistribution through descriptive meritocracy. Coefficients are standardized; total effect in parenthesis; *p < 0.001.

We tested our hypothesized mediation model (see Figure 2) using PROCESS Model 4 (bootstrapping 10.000 samples, 95% CI; Hayes 2013) and the Monte Carlo test (Yzerbyt et al. 2018) as a robustness check.^2^ Perceived economic inequality was the predictor (X), descriptive meritocracy (M) was the mediator, and attitudes towards redistribution (Y) was the outcome variable. Results showed that perceived economic inequality had an indirect effect on attitudes towards redistribution through descriptive meritocracy (*b* = .04, *SE* = .01; 95% CI [.02, .06]). As shown in Figure 2, perceived economic inequality negatively predicted descriptive meritocracy, *b* = – 0.31, *SE* = 0.03, *p* < 0.001, 95%CI [– 0.42, – 0.31], and descriptive meritocracy negatively predicted attitudes towards redistribution, *b* = –0.12, *SE* = 0.02, *p* < 0.001, 95%CI [–0.16, –0.07].

We tested the same mediation using age, gender, subjective socioeconomic status, political orientation, and preferences for meritocracy as control variables. The results were similar after controlling for them (*b* = .01, *SE* = .01; 95% CI [.001, .028]). We also found in exploratory analyses that mediation was not moderated by political orientation (see p. 6 in Supplementary material).

### Discussion

This study provides initial support for the mediation role of descriptive meritocracy in the relationship between perceived economic inequality and attitudes towards redistribution. Perceptions of economic inequality appear to challenge perceptions of meritocracy, which in turn increases support for restorative policies such as redistribution. This suggests that the effect of perceived inequality on preferences for redistribution is, at least in part, mediated by changes in perceptions of meritocracy, with higher perceived inequality leading individuals to question the fairness of the system and, consequently, to support redistributive policies.

The sample collection procedure and the contextualization of the measures within the Spanish context bolster the presumption of ecological validity. Nevertheless, it is important to acknowledge that the correlational design nature of this study inherently constrains the level of internal validity, which adds to the existing limitations in inferring causality (see Rohrer et al. 2022). Consequently, the subsequent two experiments were conducted with the aim of conceptually replicating these findings and establishing causality within the hypothesized sequence of relationships. To achieve this, we followed an experimental-causal-chain design (Spencer et al. 2005) in which we conducted two separate experiments to examine the indirect effect: a) the effect of perceived economic inequality on descriptive meritocracy, and b) the effect of descriptive meritocracy on attitudes towards redistribution.

## Study 2a. Causal effect of perceived economic inequality on descriptive meritocracy

### Methods

#### Participants

We conducted a priori sample size analysis using G*Power (Faul et al., 2009) to detect a medium effect size (*d* = .36)^3^ with 80% power and an *α* of .05 for testing independent samples t-test. Results showed that we needed 194 participants. We recruited participants via academic mailing lists and excluded those under 18 years old or who did not report their age (*n* = 1), those who failed the attention check (*n* = 1), and those who, being not native speakers, had not been living in Spain in the last 5 years (*n* = 4). The final sample size was 314 (*M_age_* = 29.27, *SD_age_* = 12.56; Women = 71.97%). A sensitivity power analysis revealed that our sample size could detect an effect size of *d* = .28 (*α =* .05*, power =* 80%). Participants agreed to voluntarily participate in a study on social issues through an online Qualtrics survey. In exchange for their participation, respondents were eligible for a €50 prize in a raffle. This experiment was preregistered in OSF: https://osf.io/s59z8/?view_only=a0956710e772451dbe320b6d4b7df4d7.

#### Procedure and Measures

First, we manipulated perceived economic inequality using the ‘Bimboola paradigm’ adapted by Sánchez-Rodríguez et al. (2019) from Jetten et al. (2015). Participants were asked to imagine their new life in a fictional society called Bimboola. Bimboola is divided into three income groups (wealthy, middle, and poor), and participants were all assigned to the middle-income group earning 7,000 Bimbolean Coins (BC) per month. They were then randomly placed in either the high inequality condition (*N =* 162), where income differences were large (wealthy: 13,500 BC; poor: 500 BC), or the low-inequality condition (*N =* 152), where income differences were minor (wealthy: 8,000 BC; poor: 6,000 BC). To enhance realism, participants were asked to imagine their lives in Bimboola, and they had to choose essentials (e.g., a house, transportation, holiday) that matched their income level. While the middle group’s options were identical across conditions, the wealthiest and poorest groups had markedly different options: in the high inequality condition, the wealthiest had access to extravagant items, while the poorest were limited to substandard goods and could not afford holidays, unlike in the low-inequality condition.

Following the manipulation, participants completed the manipulation check, which was an adaptation to Bimboola of the perceived economic inequality measure used in Study 1 (e.g., ‘To what extent is Bimboola unequal?’, *Spearman-Brown* ρ = .95). Answers were provided on a 9-point Likert scale ranging from 1 (*Not at all*) to 9 (*Completely*). Next, we measured descriptive meritocracy with the 3-items used in Study 1 adapted to Bimboola (e.g. ‘In Bimboola, people are rewarded for their efforts’, *α* = .85). Following the rationale of Study 1, we also measured subjective socioeconomic status, political orientation, and preferences for meritocracy in Bimboola as control variables.

### Results and Discussion

Our manipulation was shown to be successful as participants in the high inequality condition (*M* = 7.96, *SD* = 1.51) perceived significantly more inequality than participants in the low inequality condition (*M* = 4.82, *SD* = 1.81), *t*(295) = –16.60, *p* < 0.001, *d* = –1.88.

**Figure 3:**
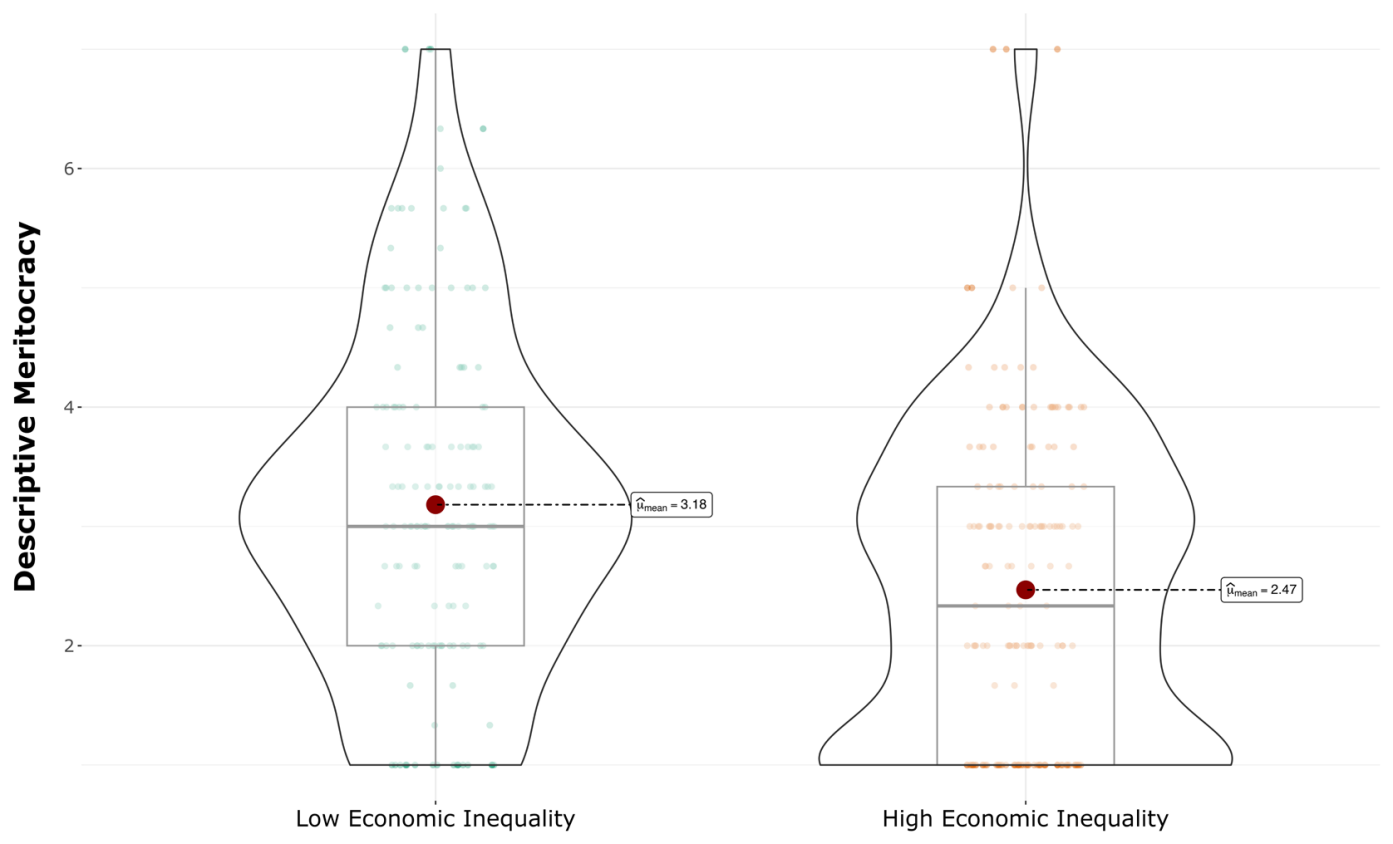
Effect of perceived economic inequality on descriptive meritocracy.

As hypothesized in the preregistration, participants in the high inequality condition perceived significantly less meritocracy in Bimboola (*M* = 2.46, *SD* = 1.34) than participants in the low inequality condition (*M* = 3.18, *SD* = 1.45), *t*(306) = 4.59, *p* < 0.001, *d* = .52 (see Figure 3). After controlling for sex, age, subjective socioeconomic status, political orientation, and preferences for meritocracy in Bimboola, the main effect of perceived inequality remained, *F*(1, 306) = 23.26, *p* < 0.001, *d* = .27.

These results serve as a conceptual replication of the findings from Study 1 regarding the first path of the indirect effect, as well as a replication of the results from Zhu et al.’s (2022) Study 2a within the Spanish context. In addition, they strengthen the internal validity of the model by revealing the causal effect of perceived economic inequality on descriptive meritocracy. To complete the experimental causal chain, we test the causal relation between descriptive meritocracy and attitudes towards redistribution shown with a correlational design in Study 1.

## Study 2b. Causal effect of descriptive meritocracy on Attitudes towards redistribution

### Methods

#### Participants

Following the same a priori sample size analysis of Study 1, we estimated that we needed 194 participants to detect a medium effect size (*d* = .36) with 80% power and an *α* of .05 for testing independent samples t-test. We recruited participants via southern university mailing lists and social networks. Due to an incorrect setting in the response recording system, many incomplete responses were recorded, so we did not include participants who did not complete the survey up to the main dependent variable, the measure of attitudes towards redistribution (n = 94). In addition, following the pre-registered criteria, we excluded those participants under 18 years old or who did not report their age (*n* = 17) and those who, being non-native speakers, had not been living in Spain for the last 5 years (*n* = 1). The final sample size was 216 (*M_age_* = 48.30, *SD_age_* = 12.06; Women = 59.26%). Participants agreed to voluntarily participate in a study about social perceptions and attitudes towards political issues through an online Qualtrics survey. This experiment was preregistered in OSF: https://osf.io/vh8xa/?view_only=52fd42f520ce486e869a47bb1fe222c4.

#### Procedure and Measures

We manipulated descriptive meritocracy using an adaptation of the ‘Bimboola paradigm’ used by Sommet & Elliot (2022) to manipulate perceived competitiveness. Participants were randomly assigned to one of two conditions: high (*N =* 105) vs. low (*N =* 111) perceived meritocracy condition. As in the Bimboola paradigm, they first were asked to imagine their new life in a fictional society, now called Mericia. They were first informed of the results of a poll conducted in Mericia in which Merician citizens were asked ‘to what extent hard work and talent are rewarded? (1 = *Not rewarded at all*, 7 = *Fully rewarded*)’. We present participants with the mean (fictitious) response of Merician citizens to this question: In the high meritocracy condition the mean fictitious response score to this question was considerably higher (6.53 out of 7) than in the low meritocracy condition (1.71 out of 7). Next, participants were told they were going to meet Merician citizens. In the high meritocracy condition, participants read five extracts of conversations with citizens of Mericia (e.g., ‘In Mericia, only people who work hard and/or have talent achieve success’). In the low meritocracy condition, these extracts were written to elicit a low meritocracy context (e.g., ‘In Mericia, only the lucky ones or those who come from wealthy families achieve success’). For a detailed description of the manipulation, see pp. 16–23 in Supplementary material.

As a manipulation check, participants completed the descriptive meritocracy measure used in Study 2a adapted to Mericia (e.g., ‘In Mericia, people are rewarded for their efforts’, *α* = .92, *M =* 3.44, *SD =* 2.13). Next, attitudes towards redistribution were assessed, adapting to Mericia the same measure used in Study 1 (e.g., ‘The government of Mericia has a responsibility to reduce the income gap between those who have more and those who have less’, *α* = .86, *M =* 5.26, *SD =* 1.39). As in Studies 1 and 2a, we measured subjective socioeconomic status and political orientation as control variables. To test non-focal ideas, in this study we include a measure of preferences for meritocracy (see p. 24 in Supplementary material) different from the one used in the previous studies.

### Results and Discussion

Our manipulation was revealed to be effective, as participants in the high meritocracy condition (*M* = 5.22, *SD* = 1.40) perceived significantly more meritocracy than participants in the low meritocracy condition (*M* = 1.75, *SD* = 1.05), *t*(192) = –20.47, *p* < 0.001, *d* = –2.80.

**Figure 4:**
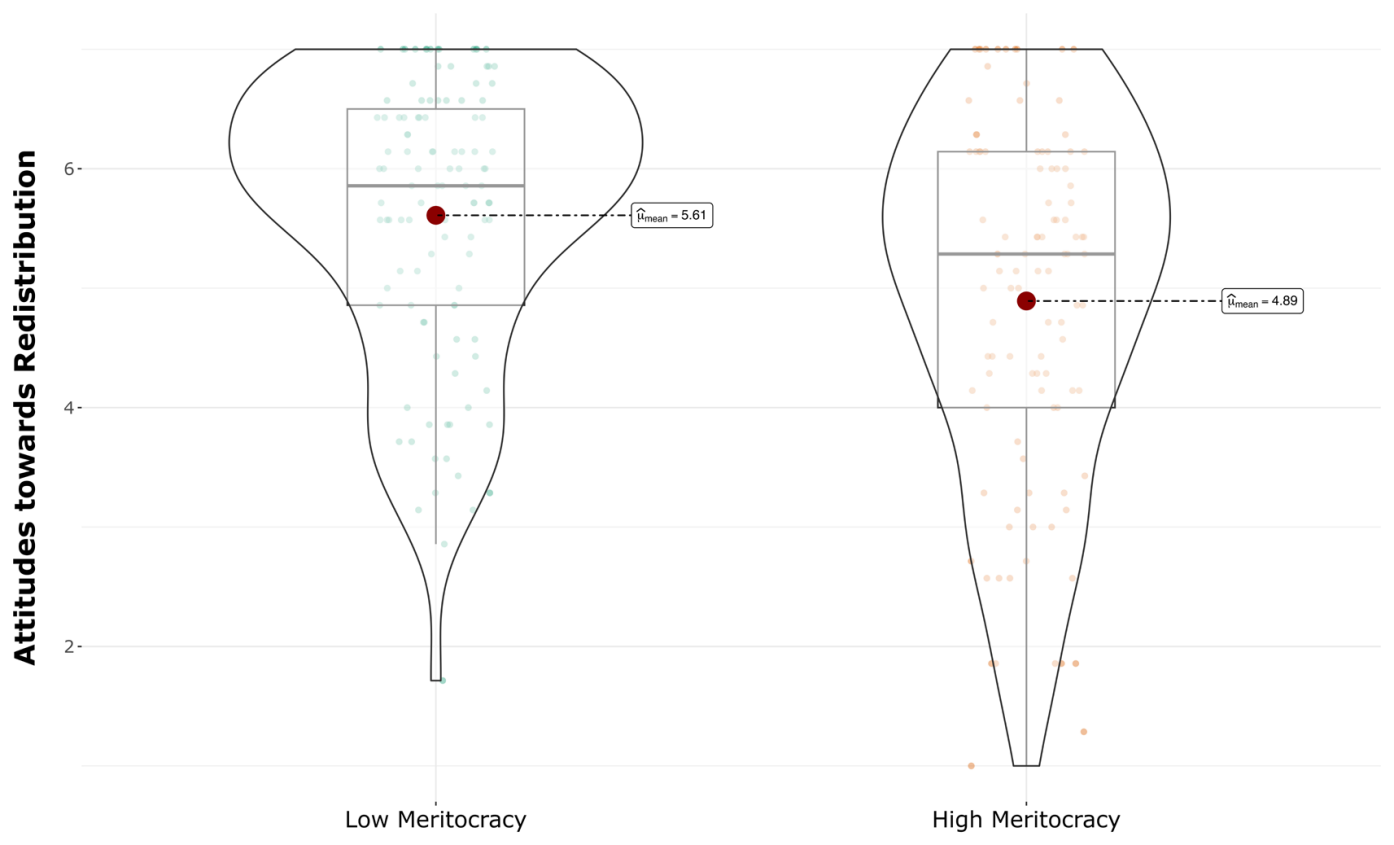
Effect of descriptive meritocracy on attitudes towards redistribution.

As hypothesized, participants in the low meritocracy condition showed more positive attitudes towards redistribution (*M* = 5.61, *SE* = 1.17) than participants in high meritocracy condition (*M* = 4.89, *SE* = 1.51), *t*(196) = 3.88, *p* < .001, *d* = .53, Figure 4). After controlling for sex, age, subjective socioeconomic status, political orientation, and preferences for meritocracy, the results remained the same, *F*(1, 203) = 18.24, *p* < 0.001, *d* = .29.

These results completed the experimental-causal-chain design, serving as a conceptual replication of the findings from Study 1 regarding the second path of the hypothesized indirect effect. In addition, they strengthen the internal validity of the model by examining the causal effect of perceived economic inequality on descriptive meritocracy.

## General Discussion

In this research, we examined the role of descriptive meritocracy in the relationship between perceived economic inequality and support for redistribution using both correlational (Study 1) and experimental approaches (Studies 2a and 2b). Study 1 revealed that the perception of economic inequality significantly predicts attitudes towards redistribution in the Spanish context in line with previous research (e.g., García-Castro et al. 2022). Importantly, we found that this relationship is partially explained by participants’ perception of meritocracy. Specifically, a higher perception of economic inequality predicted a lower perception of meritocracy within Spain, which, in turn, led to more favorable attitudes towards redistribution. Therefore, Study 1 provided cross-sectional support for our hypothesis while maintaining ecological validity.

Studies 2a and 2b replicated the indirect effect identified in Study 1 following an experimental-causal-chain design, thereby enhancing internal validity. In Study 2a, we demonstrated that the manipulation of the perception of economic inequality influences the perception of meritocracy. Specifically, the perception of economic inequality triggered a decreased perception of meritocracy. In Study 2b, we further established the paths causality by manipulating descriptive meritocracy. We showed that low perception of meritocracy predicts more favorable attitudes towards redistribution.

This research makes three fundamental contributions that are interspersed with important insights. First and foremost, it shows the role of descriptive meritocracy as an explanatory mechanism for the relationship between perceived economic inequality and support for redistribution. While prior studies have explored how inequality perception influences support for redistribution through factors such as intolerance towards inequality (García-Castro et al. 2020) and ideal levels of inequality (García-Sánchez et al., 2018), the mediating role of normative perception processes, as proposed by the EINIM (Sánchez-Rodríguez et al. 2023), remained overlooked. This research represents an important step forward by shedding light on the specific role of descriptive meritocracy.

Furthermore, it introduces a novel perspective on the study of meritocracy when considering the consequences of inequality, highlighting its mediating role when conceiving it as a descriptive social norm. It is important to note that our research does not question the potential moderating role of meritocracy as an ideology in the effects of inequality, as suggested by previous research (Batruch et al. 2022; García-Sánchez et al. 2019; Trump 2020). We acknowledge that meritocracy, when conceptualized as an ideology, may moderate the consequences of inequality. However, our contribution is to highlight the role of meritocracy as a descriptive norm in understanding the effects of inequality, offering new insights to the existing literature. While economic inequality may foster a more competitive and individualistic environment (Sánchez-Rodríguez et al. 2019), our findings indicate that it also challenges the perception that society is meritocratic. This questioning of meritocracy contributes to increased support for redistributive policies. Although competitive norms may have the opposite effect, potentially reducing support for redistribution, our results suggest that the perception of inequality also triggers responses aimed at addressing it by questioning the merit of the wealthiest.

Second, we replicated the effects of perceived economic inequality on descriptive meritocracy through Study 2a, where we replicated Zhu et al.’s (2022) results using the same experimental paradigm with a Spanish sample. Notably, we extend this replication by using the descriptive measure of meritocracy developed by Castillo et al. (2019). This measure not only considers merit in terms of hard work but also incorporates the concept of talent, which is consistent with Young’s (1958) definition of merit. By using this comprehensive measure, our research supports the notion that higher perception of economic inequality leads participants to lower the perception that individuals are rewarded for both their hard work and their talent (see p. 16 in Supplementary material for a separate analysis of these components). In other words, perceptions of economic inequality contribute to perceptions of a normative climate that lacks meritocracy. Thus, our findings support the existing evidence that perceptions of economic inequality shape our inferences about the norms prevalent in society (Sánchez-Rodríguez et al. 2023).

It is reasonable to inquire about the role of actual inequality in this model and the comparison of these results with previous studies on the relationship between actual inequality and meritocracy. The preliminary results of Study 1 provide valuable insights into this question, revealing a positive relationship between actual inequality and descriptive meritocracy, consistent with Mijs (2021). These results stand in contrast to the negative relationship between perceived economic inequality and the perception of meritocracy. This discrepancy highlights the importance of understanding economic inequality perception, as several of its effects depend on how it is perceived (Willis et al. 2022). Conversely, the effects of objective economic inequality may be accompanied by additional forces that inhibit or alter their impact (e.g., greater segregation). In fact, our findings in Study 1 show that objective and perceived economic inequality are not related, consistent with previous research that has shown a high degree of misperception of economic inequality (Gimpelson & Treisman 2018). This underscores the importance of not only considering the effects of objective economic inequality but also addressing how it is perceived and the effects that perception generates.

Third, our research experimentally replicates the effects of meritocracy perception on support for redistribution (García-Sánchez et al. 2020; Rodríguez-Bailón et al. 2017). It is worth noting that previous research has predominantly relied on priming tasks when manipulating meritocracy (Madeira et al. 2019). However, the replicability of the effects of such tasks has been questioned (Sherman & Rivers 2021). To address this limitation and establish the causal effect of our mediating variable (Pirlott & MacKinnon 2016), we introduced an effective manipulation of descriptive meritocracy. Inspired by Sommet and Elliot’s (2022) manipulation of perceived competitiveness, we tailored our manipulation to capture the effect of descriptive meritocratic norms. This approach allowed us to demonstrate the causal impact of the non-meritocratic normative climate on attitudes towards redistribution. Thereby, the research adds evidence of the effect of the normative climate on our attitudes related to inequality (Sánchez-Rodríguez et al. 2023).

### Limitations and Future Directions

Despite its strengths, several limitations of this research should be acknowledged, encompassing both general research limitations and those specific to the studies. First, this research was conducted in Spain, a context characterized by higher support for redistribution compared to many other European regimes, and where endorsement of meritocracy appears to have declined in recent years (Kudrnác & Pretrúsek 2023). Moreover, Spain is known for its loose normative culture (Eriksson et al. 2021), which implies that adherence to perceived norms is relatively flexible compared to countries with tighter normative settings. It is required to replicate these effects in different countries to assess the generalizability and robustness of our findings beyond the specific context of Spain.

Second, two limitations concerning the manipulations used should be acknowledged: the absence of ecological validity and the absence of a control group. On the one hand, although the first study preserves ecological validity, this is diminished in the two subsequent experiments. The two different experimental manipulations used in this paper rely on paradigms involving fictitious societies, where participants are exposed to an artificial context. While these types of manipulations are effective in altering societal constructs that are challenging to modify in the real world, they limit the generalizability of results to ecological settings and might elicit a demand effect in participants. However, given that we replicated the same effects with designs with (Studies 2a and 2b) and without (Study 1) these limitations, the results seem robust. On the other hand, the absence of a control condition limits the precise interpretation of the results, particularly in Study 2b. Without a control group for the meritocracy manipulation, it remains unclear whether the observed effect is driven by an increase of the support for redistribution in the low meritocracy condition, a decrease of the support for redistribution in the high meritocracy condition, or both. The experimental design presented participants with two highly polarized conditions regarding meritocracy perception, which may have amplified the effect, particularly in the low meritocracy condition. A control group with a more moderate representation of meritocracy would have allowed for a more precise interpretation of the directionality of this effect. Future research should aim to replicate these findings while addressing these limitations.

Third, it is important to acknowledge that this research includes a measure of preferences for meritocracy as a control variable in the three studies, but no confirmatory hypotheses were formulated in this regard. The preferences for the meritocracy measure used in Studies 1 and 2a were developed by Castillo et al. (2019), and it originally aimed to capture the prescriptive component of meritocracy. However, while it is consistent with preferences for meritocracy, we believe that it may not fully capture the prescriptive meritocratic norm. While descriptive meritocracy refers to the perception that society currently operates based on merit, prescriptive meritocracy reflects the belief that society should operate this way. In line with classic literature on social norms (Cialdini et al. 1991), we suggest that a comprehensive measure should assess not only the preference for its existence (i.e., recognizing it as desirable) but also the endorsement of the belief that it should exist (i.e., prescribing its existence). As an exploratory endeavor, Study 2b includes a measure from Castillo et al. (2021) that is more consistent with this last requirement. Nevertheless, due to the absence of confirmatory hypotheses in this regard, we refrain from elucidating the role of prescriptive meritocracy. Therefore, the examination of the role of prescriptive meritocracy in the link between perceptions of economic inequality and support for redistribution remains an important avenue for future research.

Finally, while this work emphasizes the importance of addressing perceptions of economic inequality, its practical implications are limited. Targeting perceptions alone may fail to address the broader structural issue of economic inequality, as an exclusive focus on subjective measures risks overlooking the policy relevance of objective conditions. However, we must note that the main objective of our research is to unravel the mechanisms linking perceptions of economic inequality with support for redistribution, in which, according to our results, the perception of meritocracy plays an important role. While the study of perceptions provides valuable insights into psychological processes, future research should transfer these insights into how structural processes affect support for redistributive policies, positioning them within the complex societal interactions and thus offering more refined guidance to policymakers.

## Conclusion

In this research, we add to the body of studies that seek to unravel the processes through which economic inequality affects attitudes towards policies aimed at reducing it. We contribute by showing that support for redistribution induced by perceived economic inequality is partly explained by inferences that the normative climate in unequal contexts is low meritocratic.

## Footnotes

In Study 1, the employed measures were integrated into a broader survey encompassing a diverse set of variables as part of a project that involved other researchers. Each contributing researcher within this collaborative project incorporated specific measures aligned with the focus of their investigations. We present only the measures concerning the present study.

Given that the bias-corrected bootstrap used in our analyses can have the type I error rate substantially inflated, we conducted a robustness check to confirm the magnitude and confidence interval of the indirect effect with the Monte Carlo method. Consistent with the bias-corrected bootstrap method, the Monte Carlo confidence interval for the indirect effect did not contain the 0, 95%CI[0.02; 0.06].

Following the guidelines provided by Lovakov & Agadullina (2021), we used a *d* = .36 to detect an effect size considered medium in the field of social psychology.
